# Explaining Consumers' Intention for Traceable Pork regarding Animal Disease: The Role of Food Safety Concern, Risk Perception, Trust, and Habit

**DOI:** 10.1155/2020/8831356

**Published:** 2020-10-29

**Authors:** Huy Duc Dang, Giang Thanh Tran

**Affiliations:** Economics Faculty, Nong Lam University, Ho Chi Minh, Vietnam

## Abstract

*Purpose*. The aim of this paper is to explain a consumers' intention for traceable food in the context of the African Swine Fever (ASF) outbreak, in order to provide scientific knowledge for the government's intervention to mitigate the perceived risk and to promote the development of traceable food. *Methodology*. This research employed an extended theory of planned behavior (TPB) model in predicting purchase intention/attitude toward traceable pork. The structural equation analysis (SEM) was used on a sample of 230 students in Vietnam. *Findings*. The current context of food safety issues, as well as animal disease outbreak, is beneficial to direct consumption toward traceable products. Heterogeneous impacts of trust were confirmed on how consumers perceived risks associated with the ASF outbreak. Consumers' habits of shopping places and looking for the product origin incite the positive attitude toward traceable pork. Food safety concerns also promoted a positive purchase attitude. *Originality/Value*. The study's objective is first to equip knowledge regarding the consumers' intention toward traceable food under the impact of animal disease, particularly in the context of food safety issues in Vietnam. Extended knowledge promotes tailored policies to regain consumers' confidence and facilitate the development of traceable food.

## 1. Introduction

The outbreaks of animal diseases such as bovine spongiform encephalopathy (BSE), foot and mouth disease (FMD), avian influenza (AI), and chronic wasting disease (CWD) have shown significant adverse effects on not only human health but also the economy and the society [1–4]. As animal health issues interrelate to human health, subsequent consumer' concerns about food hazards are also augmented [1]. Regardless of the government's effort to prevent and control the spread of the outbreak, as well as recover consumers' confidence, there has been a gap in the scientific knowledge in policy applications regarding animal health and food safety [1]. While traceable food arises as a potential solution to food safety issues in Vietnam [5], limited studies investigate consumers' purchase intention toward traceable food in parallel contexts of animal disease outbreak and prolong food safety issues. For that reason, this study sets out to fill this gap and contribute to the current body of literature, particularly in identifying the antecedents affecting a consumers' intention toward traceable food in these specific contexts, using the prominent theory of behavior change—the theory of planned behavior (TPB). The contribution is expected to be significant and unique in a way that it would be useful for generalization on consumption behaviors in other constrained and analogous contexts to equip practitioners such as food marketers and policy-makers with proper insights to foster the development of traceable food.

### 1.1. Theory of Planned Behavior

In general, the TPB is widely adopted as an effective tool in predicting consumers' intentions and behaviors, especially in the field of food research [6–10]. Mentioned studies contributed to extend the TPB model including variables such as risk perception and trust in food safety information [7, 8], habits, and trust in the context of food traceability [9, 10]. Mentioned factors and the TPB model were proven useful in predicting intention and self-reported purchase behavior. For that reason, this research is aimed at broadening the knowledge in the context of the African Swine Fever (ASF) disease outbreak to further explain consumers' intention to adopt traceable food in that setting.

Subjective norm, perceived behavioral control, and attitude are included in the original set of the TPB model [11]. The impacts of these antecedents on purchase intentions are often found significantly positive [9, 10]. However, it is worth noting that in studies where the influence of subjective norms is strong, the impact of perceived behavioral control deems overwhelming or nonsignificant [12, 13] and vice versa [6]. Nonetheless, this study hypothesizes that the impacts of the core set of determinants of the TPB on purchase intentions are positive and significant.

H1: Subjective norm has a significantly positive effect on intention.

H2: Perceived behavioral control has a significantly positive effect on intention.

H3: Attitude has a significantly positive effect on intention.

In the context of disease outbreak, studies have confirmed that risk perception has a negative influence on consumer purchase intention [14]. Similarly found by Lobb et al. [7], consumers cut down on their chicken consumption in the presence of risks due to the avian flu. The more consumers perceive food risks, the more likely they will act to minimize risks perceived, which can be through buying products with quality assurance from reliable sources [5, 6, 15]. In the case of traditional pork, risk perception would show a negative influence on intention to buy in the presence of food incidents [16]. However, as traceable food is likely to be understood as a safer option [5], the impact of perceived risks should promote purchase intention and thus derive positive influence.

H4: Perceived risk has a significantly positive effect on intention.

Next, trust is one of the most important determinants driving purchase intention [17] and influencing consumers' perception about animal disease risks [3]. However, studies that assess the effect of different types of trust on human health risk perceptions about animal diseases remain scant [3]. Previous studies have reported that trust in information provided by the media, especially on negative news and food scandals, lessens purchase intention toward general food [8], however, directing consumption toward safer options such as safe vegetables [18] and traceable meat [9, 17]. While a plethora of researches have exploited the direct impact of trust on purchase intention [3, 8–10] and the moderating role of trust [12], limited literature studies the indirect role of trust on purchase intention, particularly through risk perceptions. Higher degrees of trust would lead to lower levels of perceived risk and thus greater intention to purchase [9]. Trust in the effectiveness of traceability systems results in less risk perceived, hence prompting more purchase intentions [10, 19]. As seen in Chen and Huang [20], traceability practices of the fast-food stores help to reduce consumers' risk perceptions, thus growing the intentions for fast-food products. Risk behaviors of consumers are affected by their trust in information and the sources of that information [7, 21], as well as the actors who provided the information [7, 8, 19, 22]. Because consumers receive and evaluate food safety information from various sources, the relationship between trust and risk perception depends on the subject or information sources that consumers place their trust in [15, 19]. In the case of the escalated food safety concerns, the media could amplify the risk perceived depending on the level of media attention [23] and the frequency of the negative information acquired [24]. Thus, this study sets out to test the relationship between trust in different actors of the food chains, the traceable product, and the media on risk perceptions regarding animal diseases. In general, the risk-mitigating effect of trust, particularly on traceable products, is reasonably expected (see [17, 25]). However, the impacts of trust in different food chain actors on risk perceptions vary depending on culture and food contexts, as seen in the case of the U.S., Canada, and Japan consumers (see [3]). In the context of food safety issues repeatedly reported in Vietnam, trust in the government is weakened [2, 5, 24]. On the other hand, the positive evaluation of consumers on traceable products [5, 12] suggests that consumers might also place their trusts on the large and reputable manufacturers/food-chain operators (e.g., Vissan and CP) and retailers (e.g., Coopmart, BigC, and Lotte Mart) delivering the products, whereas their negative views on the role of the government and farmers due to food safety issues were well-reported [2, 5, 24, 26]. Because of the high frequency of negative information acquired from the media regarding food safety incidences, it is reasonable to expect that consumers who trust the media are likely to be more aware of the increasing level of food risks. For the mentioned reasons, we hypothesize that trust in manufacturers and retailers has a negative effect on risk perception, while trust in the product, the media, and the government facilitates more risks perceived.

H5: Trust in the product has a significantly positive effect on risk perception.

H6: Trust in the manufacturers has a significantly negative effect on risk perception.

H7: Trust in the government has a significantly positive effect on risk perception.

H8: Trust in farmers has a significantly positive effect on risk perception.

H9: Trust in the media has a significantly positive effect on risk perception.

H10: Trust in the retailers has a significantly negative effect on risk perception.

The relationship between risk perception, attitude, and intention was studied [8]. In a common sense, risk perception has a negative impact on attitude regarding common foods [8, 14]. Nevertheless, we expect a positive effect of risk perception on attitude toward traceable foods, which act as a risk-mitigating option. The more risk perceived, the more likely consumers could express a positive attitude toward traceable foods. Thus, this paper hypothesizes that risk perception impacts positively on attitude toward traceable pork.

H11: Risk perception has a significantly positive effect on attitude.

Additionally, habits were shown as one of the crucial factors influencing consumption behaviors, especially final decision-making regarding healthy food consumption [9, 10, 27, 28]. The motivation for purchasing the traceable chicken and honey in Italy was shown to connect with the habits of looking for specific information on a product, especially the country of origin [9]. In the case of purchase intention toward traceable minced beef and beef steak in England, the production process habits and origin habits were demonstrated to be of higher impact on intention than the perceived behavioral control (PBC) element [10]. Although mentioned studies have successfully confirmed the role of habits in various extended TPB models, experts recommend that independent measures of habits should be in place [9]. And because habits are defined as a psychological factor including both repetition and automaticity [9, 27], positive habits prior to influencing consumers' purchase intention must have already anchored down positive effects on consumers' attitude in order to make the whole process automatic. Thus, we presume that habits as well affect attitude toward traceable food and follow the suggestion of Menozzi et al. [9] and expand from the research of Spence et al. [10]; this paper hypothesizes the positive effect of four types of habits on consumers' attitude toward traceable pork.

H12: Food assurance habits have a significantly positive effect on attitude.

H13: Production process habits have a significantly positive effect on attitude.

H14: Origin habits have a significantly positive effect on attitude.

H15: Shopping place habits have a significantly positive effect on attitude.

**Figure 1:**
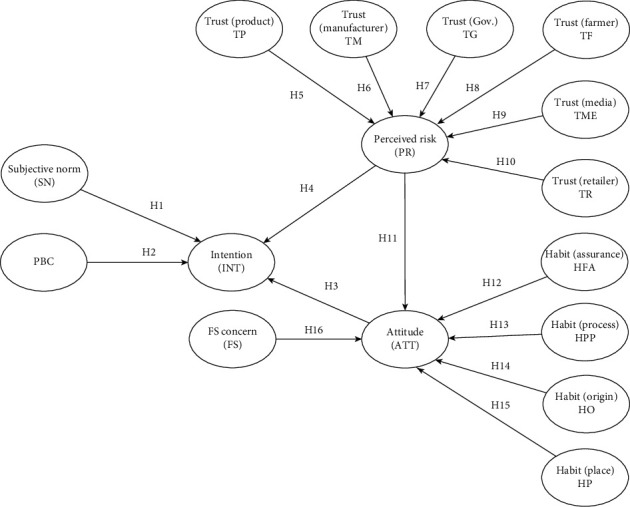
Conceptual framework.

Last, most studies assume that food safety concern in developing countries is low compared to developed countries due to the fact that consumers in developing countries are less exposed to the information regarding food hazards and risks [29]. However, recent studies in Vietnam contend that consumers do care about food safety issues and even at a high level [5, 24, 30, 31]. Based on the impressive number of smart phone users in Vietnam (84% of Vietnam population as of 2017), the above assumption of media underexposure is far from the reality. Since 2017, Ho Chi Minh City (HCMC) has been proactively promoting the development and exposure of the application of food traceability system for pork, chicken, and egg via Te-food system (see Figure 1). Hence, we hypothesize that consumers' food safety concern would likely enhance their attitude toward traceable pork.

H16: Food safety concern has a significantly positive effect on attitude.

The conceptual model can be seen in Figure 1.

## 2. Methodology

### 2.1. Data Collection and Sampling Description

This study employed a cross-sectional survey. The authors used the stratified random sampling method to collect data through face-to-face interviews using a structured questionnaire during June 2019 amid the outbreak of ASF. The respondents remained anonymous without their names and contact information recorded. Participation consent was first obtained. Those who wish not to undertake the survey were dismissed. This ruled out the bias of coercive interview which is likely to result in false information. No remuneration was made to the interviewees. The average completion time for the questionnaire was about 20 minutes. All completed questionnaires were scanned to assure no missing data. Data were collected from the students on campus. Even though the student sample may not represent the entire population, they are considered a suitable target due to their frequent use of smart phone which facilitates the adoption of traceable food through QR code scan [17].

Overall, the study sample consists of 230 students (81 male and 149 female) from Nong Lam University, in HCMC. Following the minimum*R*^2^value method, four components were identified including the minimum*R*^2^values to be detected of approx. 0.10, the significance level of 1%, and assuming the commonly used level of statistical power of 80%, and the maximum number of arrows pointing at a construct in the model which is six, derive the minimum required sample size of 217 ([32], pp. 20-21). Therefore, the sample size in this study satisfies the minimum requirement necessary for PLS-SEM analysis.

The majority of students are sophomores and juniors (66.95%). 68.26% earned less than 5 million VND per month. Most of them were not from HCMC (88.26%) and live alone (89.57%) either in the dorm or rented apartments. This matched the situation in most universities in HCMC where students from other provinces accounted for a much larger percent compared to local students. All were well aware of the current ASF outbreak. The descriptive statistics can be seen in Table 1.

### 2.2. Questionnaire Design and Outline

The questionnaire consisting of close-ended questions was pretested among several random students for understanding and content soundness, as well as the average duration. The first section of sociodemographic characteristics included gender, age, education attainment, monthly income, household size, origin, and year of study. Prior to the second section of related behavioral items, the participants were provided with a definition and illustrated example of traceable pork and the pork traceability system (Te-food). The second section contained items measuring trust in different subjects (product, manufacturer, farmer, retailer, and media); habitual behaviors regarding shopping places, product origin, product assurance, and the production process; food safety concern; perceived risk; subjective norm; perceived behavioral control; attitude; and intention.

### 2.3. Definition and Pictorial Illustration of Traceable Pork and the Tracking System

**Figure 2:**
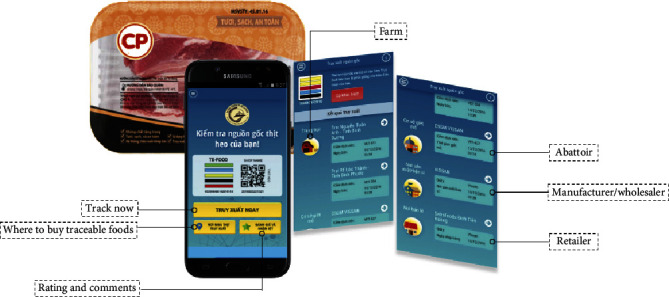
Traceable pork and Te-food app interfaces for pork.

The interviewer explained to the respondents the following definition of traceable pork: “traceable pork is different from the traditional pork available in both wet markets and the supermarkets or related food stores because it contains details of the meat regarding the entire process from farms to the retailers. The tracking process can be done by scanning a QR code on the pack via your smart phone or entering the code directly to the tracking website online. You can retrieve information about its farmers, its abattoir (name, time of slaughter, vet), manufacturer/wholesaler, retailer, and the information of the tracking company.” An illustration showing the sample of traceable pork with the Te-food app was printed on the questionnaire as shown in Figure 2.

### 2.4. Measures

Items listed in Table 2 were rated on a 7-point Likert-type scale (1 “absolutely disagree” and 7 “absolutely agree”) except for attitude with a 7-point semantic differential scale with 5 different nuances.

#### 2.4.1. Trust in Product

Following Spence et al. [10], this construct was evaluated with three statements: “I trust that traceable pork can be traced back to the actual farm,” “I trust the information provided about the production process and origin of the traceable pork,” and “I trust that traceable pork is authentic which means it has not been tampered with in any way and is what it says it is,”

#### 2.4.2. Trust in the Government/Farmers/Manufacturers/Retailers

Following Muringai and Goddard [3] and De Jonge et al. [33], these four constructs were measured by four items regarding four actors starting with the lead-in “I trust that the government/farmers/food manufacturers/retailers” accompanied by four items “[…] is/are honest about the safety of food,” “[…] is/are sufficiently open about the safety of food,” “[…] take/s good care of the safety of our food,” and “[…] give/s special attention to the safety of food.”

#### 2.4.3. Trust in Mass Media

Adapted from Muringai and Goddard [3] and De Jonge et al. [33], the construct was investigated with four items starting with the lead-in “I trust that the media” and followed by “[…] is honest about the safety of food,” “[…] is sufficiently open about the safety of food,” “[…] takes good care of the safety of our food,” and “[…] gives special attention to the safety of food,”

#### 2.4.4. Habits

Four types of habit (buying from trusted places, looking for product origin, looking for product processing information, and looking for food assurance information) were measured using the 4-item self-report behavioral automaticity index [34]: “I do automatically,” “I do without having to consciously remember,” “I start doing before I realize I am doing it,” and “I do without thinking.” Higher scores indicate stronger habit power.

#### 2.4.5. Food Safety Concern

Adapted from My et al. [30] and Michaelidou and Hassan [35], three most popular reported malpractices of pig farmers were used to measure consumers' food safety concern: “I am very concerned about the residue amount of beta-agonist (super lean substance) in pork,” “the quality of safety of pork nowadays concern me,” and “I am very concerned about the residue amount of antibiotics in pork,”

#### 2.4.6. Perceived Risk

Extended from Muringai and Goddard [3], consumers' self-report risk perception of consuming meat from ASF-infected pork was assessed with four possible consequences/symptoms: “high fever,” “intense headache,” “nausea,” “gastrointestinal toxicity,” and “meningitis.” ASF does not cause zoonotic diseases. However, ASF-contracted pigs are likely to be infected with other opportunistic diseases such as blue ear, swine flu, and typhoid fever, which can lead to mentioned symptoms once consumed.

#### 2.4.7. Subjective Norm

The perceived social influence toward buying traceable pork was analyzed with five social sources including family, partner, and friends; university scientists; the media; the food industry; and other crucial people.

#### 2.4.8. Perceived Behavioral Control

Following Spence et al. [10], this construct gauged the perception of the capability to comprehend information regarding the production process and origin of traceable pork.

#### 2.4.9. Attitude

Consumers' attitude toward buying traceable pork compared to the conventional one available in the supermarkets was evaluated by five semantic differential scales under two categories of affectional (bad-good, unpleasant-pleasant, and negative-positive) and cognitive perspective (foolish-wise and harmful-beneficial) [10, 36].

#### 2.4.10. Intention

Intention to shop traceable pork was assessed by different degrees of willingness to purchase or to increase the chance of buying.

The structural equation model (SEM) was done using the partial least square (PLS-SEM); WarpPLS 7.0 software was employed for analysis and hypotheses testing. Compared to CB-SEM, PLS-SEM gains significant advantages such as dealing with nonnormal data and small sample sizes and facilitating the use of both reflective and formative indicators [37].

## 3. Results

### 3.1. Measurements of Reliability and Validity

The first step is to evaluate the reliability and validity of the observable items used to measure constructs. All Cronbach's alpha coefficients exceed 0.7 (Table 3), indicating internal consistency reliability of the measurement scales. All items' loadings are greater than 0.5 (Table 4). Significant convergent validity is confirmed as the crossloading matrix indicates that items load more inside their designated constructs rather than with other constructs [38]. To test the discriminant validity, Table 3 exhibits that the square root of the AVE of a specific construct, on the diagonal, is larger than the correlation between it and other latent constructs. Hence, the model indicates acceptable discriminant validity [38, 39]. The threat of common method bias is also examined. Common method bias in a PLS-SEM context is often originated from the measurement method rather than from the causality assessment of the studied model [38]. One of the key reasons why this bias matters is attached to the inflation (type I errors) and deflation (type II errors) effects of path coefficients. Past studies adopt the confirmatory factor analysis (CFA) as a solution to the bias. However, models with common method bias are proven to bypass the two critical criteria of convergent and discriminant validity within the CFA [38, 40]. Mentioned authors thus proposed an alternative, the full collinearity test (FCT), which combines the classical collinearity and lateral collinearity to test both the predictor-predictor and predictor-criterion relationships. The FCT is reported to successfully identify common method bias with a combined variance inflation factor (VIF) greater than 3.3 [38]. In this study, all full collinearity VIFs are less than 2.7, indicating no problem of common method bias.

### 3.2. Structural Model Assessment

**Figure 3:**
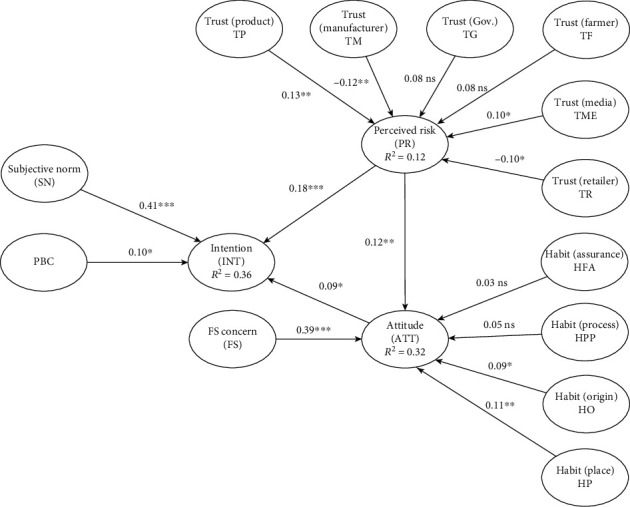
PLS results of the hypothesized model. Note: ^∗∗∗^*p* < 0.001, ^∗∗^*p* < 0.05, and ^∗^*p* < 0.1.

Model fit indices were acceptable, average path coefficient (APC) (0.136, *p* = 0.009), average *R*-squared (ARS) (0.268, *p* < 0.001), average adjusted *R*-squared (AARS) (0.250, *p* < 0.001), average block VIF (AVIF) (1.539), goodness of fit (GOF) (0.441), and standardized root mean squared residual (SRMR) (0.087) [41]. The model explains 12% of the variance for perceived risk, 32% of the variance for attitude, and 36% of the variance for intention to purchase traceable pork.  Figure 3 exhibits the tested model. The results of hypotheses testing can be found in Table 5.

Except for H7, H8, H12, and H13, the remaining hypothesized relationships were supported.

All the relationships between core constructs of the TPB and intention were supported, including subjective norm (*β* = 0.41, *p* < 0.001), perceived behavioral control (*β* = 0.10, *p* = 0.07), and attitude (*β* = 0.09, *p* = 0.07).

Regarding perceived risk, the study found its significant positive impact on intention (*β* = 0.18, *p* < 0.001). While H5 reported the positive impact of trust in the product on perceived risk (*β* = 0.13, *p* = 0.02), H7 revealed the negative impact of trust in manufacturers on perceived risk (*β* = −0.12, *p* = 0.03). The impact of trust in the media was significantly positive on perceived risk (*β* = 0.10, *p* = 0.06), whereas trust in retailers had a negative impact on perceived risk (*β* = −0.10, *p* = 0.07).

In accordance with H11, the research result supported the favorable effect of perceived risk on attitude (*β* = 0.12, *p* = 0.03). For H14 and H15, the habit constructs held positive impacts on attitude, namely, habit of checking food origin (*β* = 0.09, *p* = 0.08) and habit of purchasing at trusted places (*β* = 0.11, *p* = 0.04). Lastly, food safety concern was positively related to attitude (*β* = 0.39, *p* < 0.001).

## 4. Discussion

The aim of this paper was to test the proposed model to understand potential key determinants that affect the intention of consumers to purchase traceable pork in the context of the ASF outbreak. The results showed that the core set of antecedents of purchase intention toward traceable pork has positive impacts on intention. Notably, the impact of subjective norm outperformed that of perceived behavioral control and attitude. Indeed, in studies where the influence of subjective norm (*β* = 0.41) is strong, the impact of the other two, especially perceived behavioral control (*β* = 0.10), deems overwhelming or nonsignificant [11–13]. This study is in line with these findings.

Different consumption patterns were found in the EU after the BSE crisis, and these different reactions were found to depend on how risk was perceived [42]. In general, people tend to change their current behavior in order to protect their health and reduce the risk associated with that former behavior [4]. In the case of potentially dangerous food options, consumers' perceived risk contributed to the decrease in likelihood of purchasing, as in the case GM food in Italy and the U.S. [43], street food [14], or chicken meat amid the emergence of the avian influenza [7]. However, traceable food is perceived as a risk-mitigating option; this study thus provided evidence to support that high-level risk perception regarding the ASF outbreak would likely to promote the intention toward traceable pork.

Amid the H5N1 outbreak in the U.S., Beach et al. [43] argued that if the risk was told to be negligible by the credible public health authorities, consumers have no reason to sway their risk perception and subsequently their change of food choices. These authors also mentioned the identical shared situation observed in the BSE outbreak in 2003, where government agencies announced the associated risk negligible. This reinforces that the effect of risk perception is likely to depend on consumers' trust in trusted information sources (e.g., government health agencies). This research found the impacts of trust in product, manufacturers, media, and retailers on risk perception significant, while not for trust in the government and farmers. This deems aligned with our understanding of the perceived negative role of the government and farmers in Vietnam and in line with mentioned studies [2, 5, 24, 31]. The impacts of the extant trust constructs were supported. Because of the expensiveness and procurement requirements of traceable food, only designated food distributors—mostly large food-chain operators or the supermarkets—carry these products and the sellers are big enough that their brand names enhance the credibility of the traceable foods. Thus, consumers who trust these food distributors would likely to perceive less risk associated with animal diseases. The findings are analogous to Muringai and Goddard [3]. Furthermore, trust in the traceable pork itself, in the context of food traceability, would result in the increase of certainty and safety [17, 25]. This was possible thanks to the informativeness provided by the traceability system which reduces information asymmetry [44]. Researches have shown that risk amplification by the media is more effective when the information provided is negative rather than positive due to the trustworthiness of the negative one over the positive one [22]. Thus, a higher level of trust in the information delivered by the media is likely to build up risk perception accordingly [7]. The results of this paper agree with the mentioned findings. Contrary to the findings of Nguyen et al. [45] and Nguyen and Ngo [16] who studied the role of perceived risk on attitude toward conventional pork purchase, this paper confirmed the positive impact of perceived risk on attitude toward traceable pork. In a similar rationale, consumers who perceived a higher risk of animal diseases would adopt traceable pork as a safer option to safeguard their family health.

Regarding habit constructs, studies found distinct impacts of habits on the intention to purchase in terms of researched countries and commodities. For example, the impacts of habits of looking for information about the country of origin, production process, and certificates of traceable chicken and honey on purchase intention were found heterogeneous between France and Italy [9]. Spence et al. [10] found the positive impact of the origin habit and production process habit driving intention to purchase beef steak but not minced beef. In this study, we found the positive impacts of origin habit (habits of seeking for country or region of origin) and shopping place habit (habits of buying from trusted sources—mostly the supermarkets or convenient food stores) on attitude toward purchasing traceable pork instead of the traditional one, while the influence of assurance and production process habits were not significant predictors. Perhaps, this implies the fact that Vietnamese consumers might not be familiar with the traceability systems which are still at their early stage [5, 26]. It is also worth discussing that consumers' familiarity with traceability information as well as their attitude toward traceable food can be promoted through their trusted shopping places such as the well-known supermarkets or convenient food stores.

Regarding food safety concern, it was commonly found that the more consumers concerned about food safety issues, the more probable they will opt for traceable food options [5, 46] and willing to pay more [26, 44]. For the fact that the relationship between the food safety incidents, consumer confidence, and consumer behavior has received meager investigation [22] and in developed countries such as the EU where food traceability is mandatory, consumers are still giving a considerable amount of concern over the safety of food [47]. The role of food safety concern in shaping consumers' attitude/intention toward safe food has never been more critical, especially in the context of traceable meat in Asia [17]. In the context of long-lasting food issues, traceable food is expected to be a solution for a safer healthy eating lifestyle. This study further extends and confirms the findings of My et al. [30] and Dang et al. [5] that consumers worrying about food safety issues are likely to opt for traceable meat.

## 5. Conclusions, Implications, and Limitations

The study applied the SEM approach and the extended TPB in explaining consumers' behaviors for traceable pork in the context of the ASF outbreak in Vietnam. Promising results suggest that consumers who perceived the risk of consuming infected pork or the concern over the safety of overall pork tend to embrace a positive attitude/intention toward traceable pork. Trust in different food-chain actors, the product, and the media exhibits a heterogeneous impact on how consumers perceive risk. To promote consumption toward traceable pork, a possible policy implication is to address and expose misconduct and bad practices of pig rearing and processing as well as farming and amplify the risk perceiving effect through the media. Moreover, traceable food should continue to be distributed by trusted sellers. To create synergy with trust, traceable food distributors might also want to target popular go-to meat shopping locations and educate the consumers about the transparent origin of the products because of the positive effect of their origin habit and shopping place habit on attitude toward traceable food. This paper is unique in extending literature about the impact of the current food safety concern toward traceable food in the context of rampant animal disease. Besides the perceived risk regarding the disease outbreak, consumers' food safety concerns can act as a standalone promoter to make consumers a convert of traceable food. For the mentioned analysis, we support and encourage the development of traceable food as a risk-mitigating solution in the current situation. We also suggest that the government should hurry on making food traceability compulsory similar to other developed countries. When the demand for traceable food can reach its peak, the economies of scale can certainly draw price back to a reasonable and irresistible threshold similar to that of regular pork.

We acknowledge several limitations of the research. First of all, the sample of students in our paper may not be representative of the consumer population in Vietnam. Moreover, different populations researched at different points in time will illustrate different risk perceptions, thus, dissimilar food behaviors [4]. Thus, the results of this work should be interpreted with caution, particularly for population other than students. This also opens up future research possibilities for different populations. Secondly, the difference between self-reported behaviors and real behaviors might make it puzzling to generalize the research results. Finally, despite the fact that extended TPB is useful in predicting behavior toward traceable food, the model in this study can only explain 36% of the variance of the purchase intention, which signals more work to be done. We, therefore, call for more efforts spent on studying the antecedents of purchase intention toward traceable food in similar or other contexts.

## Figures and Tables

**Table 1:** Descriptive statistics.

Variable	Categories	Frequency	Percent
Gender	Male	81	35.22
	Female	149	64.78

Student	Freshman	35	15.21
	Sophomore	76	33.04
	Junior	78	33.91
	Senior	41	17.82

Monthly income	<5 m. VND	157	68.26
	5–9.9 m. VND	33	14.34
	10-14.9 m. VND	21	9.13
	>15 m. VND	19	8.26

Household size	1 per.	206	89.57
	2 per.	2	0.87
	3 per.	7	3.04
	4 per.	12	5.22
	5 per.	3	1.30

Origin	Ho Chi Minh City	27	11.74
	Otherwise	203	88.26

**Table 2:** Constructs with items.

*Trust in product (TP)*	I trust
TP1	That traceable pork can be traced back to the actual farm.
TP2	The information provided about the production process and origin of the traceable pork.
TP3	Traceable pork is authentic which means it has not been tampered with in any way and is what it says it is.
*Trust in the government (TG)*	I trust that the government
TG1	Is honest about the safety of food.
TG2	Is sufficiently open about the safety of food.
TG3	Takes good care of the safety of our food.
TG4	Gives special attention to the safety of food.
*Trust in farmers (TF)*	I trust that farmers
TF1	Are honest about the safety of food.
TF2	Are sufficiently open about the safety of food.
TF3	Take good care of the safety of our food.
TF4	Give special attention to the safety of food.
*Trust in manufacturers (TM)*	I trust that food manufacturers
TM1	Are honest about the safety of food.
TM2	Are sufficiently open about the safety of food.
TM3	Take good care of the safety of our food.
TM4	Give special attention to the safety of food.
*Trust in food retailers (TR)*	I trust that food retailers
TR1	Are honest about the safety of food.
TR2	Are sufficiently open about the safety of food.
TR3	Take good care of the safety of our food.
TR4	Give special attention to the safety of food.
*Trust in mass media (TME)*	I trust that the mass media
TME1	Is honest about the safety of food.
TME2	Is sufficiently open about the safety of food.
TME3	Promotes the safety of our food.
TME4	Notifies audiences about food safety incidence on time.
*Habits of shopping places (HP)*	Buying pork from a supermarket, a convenient food store, or trusted sources is something
HP1	I do automatically.
HP2	I do without having to consciously remember.
HP3	I start doing before I realize I am doing it.
HP4	I do without thinking.
*Habits of country of origin (HO)*	When I buy pork, looking for information about the country, or region of origin is something
HO1	I do automatically.
HO2	I do without having to consciously remember.
HO3	I start doing before I realize I am doing it.
HO4	I do without thinking.
*Habits of production process (HPP)*	When I buy pork, looking for information about the production process that is needed to make the pork (e.g., feed, rearing conditions, transport, slaughter, and processing) is something
HPP1	I do automatically.
HPP2	I do without having to consciously remember.
HPP3	I start doing before I realize I am doing it.
HPP4	I do without thinking.
*Habits of food assurance (HFA)*	When I buy pork, looking for food assurance schemes such as VietGap/GAHP, or smaller “niche” schemes that are aimed at meeting particular consumer demands such as higher welfare, environmental, or organic standards, is something
HFA1	I do automatically.
HFA2	I do without having to consciously remember.
HFA3	I start doing before I realize I am doing it.
HFA4	I do without thinking.
*Food safety concern (FS)*	
FS1	I am very concerned about the residue amount of beta-agonist (super lean substance) in pork.
FS2	The quality of safety of pork nowadays concern me.
FS3	I am very concerned about the residue amount of antibiotics in pork.
*Perceived risk of consuming meat from affected animals (PR)*	I, or my family, have concerns about eating pork infected by ASF because it could cause
PR1	High fever.
PR2	Intense headache.
PR3	Nausea.
PR4	Gastrointestinal toxicity.
PR5	Meningitis.
*Subjective norm (SN)*	I would buy traceable pork because
SN1	My family, partner, and friends approve.
SN2	University scientists are in favor of it.
SN3	The media (TV, radio) are in favor of it.
SN4	The food industry and/or food supermarkets promote it.
SN5	People important to me buy this type of pork.
*Perceived behavioral control (PBC)*	Regarding the additional information about the production process and origin of traceable pork (obtained via the code)
PBC1	It will be easy to find the additional information.
PBC2	I will be confident that I will find the additional information.
PBC3	I will be able to find the additional information without help from others.
PBC4	It will be easy to understand the additional information.
PBC5	I will be confident that I will understand the additional information.
PBC6	I will be able to understand the additional information without help from others.
*Attitude (ATT)*	Buying traceable pork instead of pork now available in supermarkets would make me feel
ATT1	Bad (1)–good (7)
ATT2	Unpleasant (1)–pleasant (7)
ATT3	Foolish (1)–wise (7)
ATT4	Harmful (1)–beneficial (7)
ATT5	Negative (1)–positive (7)
*Intention (INT)*	When traceable pork becomes available
INT1	I intend to buy it.
INT2	I plan to buy it.
INT3	I will look for it.
INT4	I desire to buy it.
INT5	It will be important for me to buy it.

**Table 3:** Variance inflation, reliability measures (internal consistency), and correlations with square root of AVEs.

Construct	VIF	CR	CA	AVE	INT	SN	PBC	PR	ATT	FS	HFA	HPP	HO	HP	TP	TM	TG	TF	TME	TR
INT	1.859	0.916	0.884	0.686	0.828															
SN	1.805	0.893	0.848	0.626	0.546	0.791														
PBC	1.301	0.932	0.912	0.697	0.270	0.393	0.835													
PR	1.770	0.919	0.889	0.693	0.368	0.361	0.225	0.833												
ATT	1.313	0.907	0.872	0.663	0.308	0.304	0.084	0.335	0.814											
FS	2.241	0.940	0.904	0.839	0.539	0.440	0.162	0.601	0.429	0.916										
HFA	2.459	0.917	0.878	0.733	0.183	0.258	0.115	0.222	0.154	0.217	0.856									
HPP	2.772	0.912	0.871	0.724	0.160	0.243	0.095	0.196	0.192	0.220	0.740	0.851								
HO	2.412	0.881	0.816	0.655	0.255	0.252	0.074	0.176	0.210	0.213	0.615	0.668	0.809							
HP	1.684	0.872	0.797	0.639	0.237	0.255	0.046	0.149	0.155	0.104	0.453	0.462	0.599	0.799						
TP	1.465	0.881	0.797	0.713	0.106	0.137	0.180	0.195	0.147	0.248	0.077	0.145	0.095	0.035	0.845					
TM	2.172	0.943	0.920	0.807	0.107	0.217	0.144	-0.062	0.116	0.060	0.120	0.203	0.126	0.126	0.361	0.898				
TG	1.746	0.938	0.912	0.792	0.269	0.315	0.286	0.144	0.234	0.308	0.119	0.224	0.213	0.168	0.372	0.408	0.890			
TF	1.808	0.930	0.899	0.769	0.153	0.206	0.168	0.139	0.156	0.210	0.217	0.278	0.266	0.186	0.325	0.521	0.526	0.877		
TME	1.473	0.939	0.913	0.794	0.321	0.281	0.223	0.173	0.220	0.269	0.130	0.179	0.187	0.171	0.417	0.303	0.358	0.203	0.891	
TR	1.983	0.944	0.921	0.810	0.023	0.175	0.117	-0.075	0.034	0.023	0.074	0.136	0.119	0.126	0.329	0.662	0.367	0.462	0.310	0.900

VIF: full collinearity variance inflation factor; CR: composite reliability; CA: Cronbach's alpha; AVE: average variance extracted.

**Table 4:** Mean, standard deviation, and loadings.

Construct	Item	Mean	SD	Load	*p* value
TP	TP1	4.7	1.696	0.863	<0.001
	TP2	4.7	1.594	0.894	<0.001
	TP3	4.3	1.850	0.772	<0.001

TG	TG1	4.5	1.745	0.874	<0.001
	TG2	4.6	1.658	0.908	<0.001
	TG3	4.6	1.563	0.920	<0.001
	TG4	4.6	1.703	0.855	<0.001

TF	TF1	3.9	3.965	0.866	<0.001
	TF2	3.9	3.960	0.900	<0.001
	TF3	4.1	4.182	0.907	<0.001
	TF4	4.1	4.178	0.833	<0.001

TM	TM1	4.0	1.667	0.902	<0.001
	TM2	4.0	1.534	0.919	<0.001
	TM3	4.0	1.581	0.909	<0.001
	TM4	4.1	1.611	0.862	<0.001

TR	TR1	3.7	1.608	0.893	<0.001
	TR2	3.8	1.611	0.928	<0.001
	TR3	3.9	1.510	0.914	<0.001
	TR4	3.8	1.627	0.863	<0.001

TME	TME1	4.6	1.659	0.882	<0.001
	TME2	4.7	1.580	0.907	<0.001
	TME3	4.8	1.665	0.921	<0.001
	TME4	5.0	1.674	0.853	<0.001

HP	HP1	4.8	1.903	0.513	<0.001
	HP2	4.0	2.047	0.886	<0.001
	HP3	4.2	1.996	0.869	<0.001
	HP4	4.2	2.188	0.866	<0.001

HO	HO1	4.8	1.978	0.584	<0.001
	HO2	4.1	1.956	0.856	<0.001
	HO3	4.2	1.995	0.887	<0.001
	HO4	4.1	2.113	0.871	<0.001

HPP	HPP1	4.2	1.932	0.730	<0.001
	HPP2	3.9	1.881	0.886	<0.001
	HPP3	4.0	1.949	0.897	<0.001
	HPP4	3.9	2.111	0.879	<0.001

HFA	HFA1	4.2	2.031	0.786	<0.001
	HFA2	3.9	2.052	0.875	<0.001
	HFA3	3.9	1.918	0.897	<0.001
	HFA4	4.1	2.039	0.864	<0.001

FS	FS1	5.26	1.787	0.899	<0.001
	FS2	5.45	1.673	0.922	<0.001
	FS3	5.5	1.644	0.927	<0.001

PR	PR1	5.1	1.846	0.781	<0.001
	PR2	5.0	1.801	0.885	<0.001
	PR3	5.2	1.665	0.866	<0.001
	PR4	5.4	1.669	0.847	<0.001
	PR5	5.0	1.796	0.779	<0.001

SN	SN1	5.2	1.811	0.742	<0.001
	SN2	4.8	1.582	0.858	<0.001
	SN3	4.8	1.585	0.857	<0.001
	SN4	4.8	1.655	0.797	<0.001
	SN5	4.7	1.828	0.688	<0.001

PBC	PBC1	4.5	1.857	0.766	<0.001
	PBC2	4.4	1.816	0.842	<0.001
	PBC3	4.3	1.751	0.867	<0.001
	PBC4	4.3	1.745	0.866	<0.001
	PBC5	4.4	1.749	0.872	<0.001
	PBC6	4.3	1.968	0.791	<0.001

ATT	ATT1	5.4	1.599	0.796	<0.001
	ATT2	5.6	1.380	0.849	<0.001
	ATT3	5.7	1.323	0.841	<0.001
	ATT4	5.8	1.286	0.856	<0.001
	ATT5	5.6	1.526	0.723	<0.001

INT	INT1	4.9	1.728	0.818	<0.001
	INT2	4.9	1.596	0.869	<0.001
	INT3	5.0	1.564	0.867	<0.001
	INT4	5.2	1.632	0.856	<0.001
	INT5	5.5	1.688	0.721	<0.001

**Table 5:** Results of hypothesis investigation.

Hypotheses	Path directions	*β*	*p* value	Support
H1	SN → INT	0.41	0.001	Yes
H2	PBC → INT	0.10	0.07	Yes
H3	ATT→ INT	0.09	0.07	Yes
H4	PR → INT	0.18	0.001	Yes
H5	TP → PR	0.13	0.02	Yes
H6	TM → PR	-0.12	0.03	Yes
H7	TG → PR	0.08	0.12	No
H8	TF → PR	0.08	0.12	No
H9	TME → PR	0.10	0.06	Yes
H10	TR → PR	-0.10	0.07	Yes
H11	PR → ATT	0.12	0.03	Yes
H12	HFA → ATT	0.03	0.34	No
H13	HPP → ATT	0.05	0.22	No
H14	HO → ATT	0.09	0.08	Yes
H15	HP → ATT	0.11	0.04	Yes
H16	FS → ATT	0.39	0.001	Yes

## Data Availability

Data are available upon reasonable request.
